# Parkinson‐like early autonomic dysfunction induced by vagal application of DOPAL in rats

**DOI:** 10.1111/cns.13589

**Published:** 2021-01-21

**Authors:** Jie Sun, Chao He, Qiu‐Xin Yan, Hong‐Dan Wang, Ke‐Xin Li, Xun Sun, Yan Feng, Rong‐Rong Zha, Chang‐Peng Cui, Xue Xiong, Shan Gao, Xue Wang, Rui‐Xue Yin, Guo‐Fen Qiao, Bai‐Yan Li

**Affiliations:** ^1^ Department of Pharmacology (State‐Province Key Laboratories of Biomedicine‐Pharmaceutics of China Key Laboratory of Cardiovascular Medicine Research, Ministry of Education) College of Pharmacy Harbin Medical University Harbin China; ^2^ School of Pharmaceutical Science Sun Yat‐Sen University Shenzhen China; ^3^ School of Life Science and Technology Harbin Institute of Technology Harbin China; ^4^ Department of Biomedical Engineering School of Engineering and Technology Indiana University Purdue University Indianapolis Indianapolis Indiana USA

**Keywords:** autonomic dysfunction, DOPAL, Parkinson's disease, vagus, α‐synuclein

## Abstract

**Aim:**

To understand why autonomic failures, a common non‐motor symptom of Parkinson's disease (PD), occur earlier than typical motor disorders.

**Methods:**

Vagal application of DOPAL (3,4‐dihydroxyphenylacetaldehyde) to simulate PD‐like autonomic dysfunction and understand the connection between PD and cardiovascular dysfunction. Molecular and morphological approaches were employed to test the time‐dependent alternation of α‐synuclein aggregation and the ultrastructure changes in the heart and nodose (NG)/nucleus tractus solitarius (NTS).

**Results:**

Blood pressure (BP) and baroreflex sensitivity of DOPAL‐treated rats were significantly reduced accompanied with a time‐dependent change in orthostatic BP, consistent with altered echocardiography and cardiomyocyte mitochondrial ultrastructure. Notably, time‐dependent and collaborated changes in Mon‐/Tri‐α‐synuclein were paralleled with morphological alternation in the NG and NTS.

**Conclusion:**

These all demonstrate that early autonomic dysfunction mediated by vagal application of DOPAL highly suggests the plausible etiology of PD initiated from peripheral, rather than central site. It will provide a scientific basis for the prevention and early diagnosis of PD.

## Graphical abstract

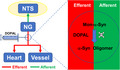

## 1 INTRODUCTION

Parkinson's disease (PD), known as the second neurodegeneration disease, is accompanied by motor disorders including resting tremor, postural instability, bradykinesia, and involuntary movements of mouth and face and non‐motor disorders including hyposmia, depression, constipation, and autonomic dysfunction.^1^, ^2^, ^3^ The most common early autonomic dysfunction is characterized by orthostatic blood pressure (BP) changes, including orthostatic hypotension (OH) and supine hypertension (SH); OH is an important cause of death in PD patients.^4^ The probability of concurrent OH in patients with PD is extremely high, and OH is a very valuable indicator for the prediction of early motor dysfunction in PD.^5^, ^6^, ^7^, ^8^ Degeneration of central dopaminergic neurons is considered to be a key factor in the pathogenesis of PD in the past few years, but recent studies have shown that the initial onset is likely to be in the periphery.^9^, ^10^, ^11^ Sacino AN et al.'s study on transgenic mice found that the pathological changes in α‐synuclein (α‐Syn, one of the major biomarkers of PD) can be found in the central nervous system (CNS) after intramuscular injection of α‐Syn, and Peelaerts W et al. also showed a similar phenomenon in adult female Wistar rats after intravenous injection of α‐Syn assemblies.^12^, ^13^ They all proved that there is a possibility that α‐Syn aggregation formed peripherally can be transported to the CNS. If so, the existing animal model of PD induced by central chemical injury cannot truly simulate the pathophysiological process and this limitation cannot be ignored. Therefore, to seek a new animal model showing a PD‐like early autonomic dysfunction by vagal administration of DOPAL and understand the potential etiology of PD initialed from peripheral are our central focus. To reveal the possible bridge communicating between the peripheral cardiovascular systems (PCVS) and CNS in the pathophysiological process of PD is also need to be clarified.

Study of PD has found that more than half (58.2%) of the patients suffered OH^14^; this is certainly not an accidental factor. OH can be divided into neurogenic hypotension and non‐neurogenic (hypovolemic) hypotension according to different factors of formation.^15^ Obviously, PD with OH does not belong to the latter. Therefore, to link PCVS with CNS, the neuroanatomical pathways will be our first choice. Vagus nerve is the 10th pair of cranial nerves which is the longest and most widely distributed in body. It mainly contains four kinds of fiber components: (1) afferent visceral sensory fibers from the heart, GI tract, lungs, and so on; (2) efferent visceral motor fibers to a similar distribution of tissues; (3) efferent somatic motor fibers to several skeletal muscles of the pharynx and larynx, and (4) somatic sensory from auricle of ear/external acoustic meatus.^16^, ^17^ And recently, Ted Dawson's study showed that misfolded α‐Syn can be transmitted from the intestine to the mouse brain through the vagus.^18^ Due to the widespread distribution of the vagus and its special identity in PD, it could be a perfect candidate to study a bridge communication between CNS and PCVS.

DOPAL (3, 4‐dihydroxyphenylacetaldehyde) plays a major role in the pathological process of PD. In the metabolic pathway of dopamine, DOPAL appears as an intermediate product, like most aldehydes, and it has strong toxicity under physiological condition when accumulates in the body.^19^, ^20^ In PD patients, DOPAL level is significantly higher than that in the healthy subjects accompanied by low activity of ALDH1A1, an aldehyde dehydrogenase (ALDH) isoenzyme that degrades DOPAL to a non‐toxic product.^21^, ^22^, ^23^, ^24^ It has well been documented that DOPAL promotes the formation of α‐Syn–aggregated oligomers and other related proteins, and indirectly exerts its neurotoxicity^25^, ^26^; furthermore, it can directly act on the mitochondria to disrupt membrane permeability, cause mitochondrial dysfunction, and trigger cell death.^27^

Based upon the evidences mentioned above, we ambitiously tried to mimic the abnormal metabolism of peripheral dopamine by direct application of DOPAL on vagus. Through this dosing model, we were able to find one or more valuable clues to understand the connection between PD and early PCVS dysfunction. It will provide a new dimension and clinical strategy for the prevention and treatment of PD.

## 2 METHODS

### 2.1 Chemicals

DOPAL (3,4‐dihydroxyphenylacetaldehyde) was purchased from Cayman Chemical Company. Stock solutions were stored at −20°C and diluted before experiments.

### 2.2 Experimental animal

All protocols about animals used in experiments were pre‐approved by Institutional Animal Care and Use Committee of Harbin Medical University, which are in accordance with the recommendations of the Panel on Euthanasia of the American Veterinary Medical Association and the National Institutes of Health publication “Guide for the Care and Use of Laboratory Animals” (http://www.nap.edu/readingroom/books/labrats/). Male Sprague Dawley (SD) rats weighing 200–250 g were purchased from the experimental animal center of the Second Affiliated Hospital of Harbin Medical University (Harbin, China; the certificate number: SCXK‐2019‐001).

### 2.3 Vagal application of DOPAL

Unrestrained adult male rats (200–250 g) were anesthetized by 3% sodium pentobarbital (25 mg/kg) intraperitoneally (i.p.). After that, rats were placed in a supine position and the neck was disinfected with 75% ethanol. A 3.0 cm incision was then made longitudinally along the midline of the neck, and all subsequent surgical procedures were conducted under stereomicroscope (40×). First, we used tweezers to laterally pull the incision to fully expose the surgical area, and then, the left nodose was carefully dissected to ensure complete isolation of the nodose from the carotid arterial and aortic depressor nerve. Finally, the DOPAL (5.0 μl, 0.8 mg/ml, dissolved in saline) was microinjected into the vagus at approximately 1.0 cm from nodose toward caudal direction using a precision micro‐syringe (Hamilton, Nevada, USA) and a 30‐G half‐inch stainless steel syringe needle with a 35° beveled tip, with which the caution is needed to not force too much or break the nerves. During the microinjection, we can clearly see a bulge at the injection site, which means that DOPAL is successfully injected into the vagus. In the sham group, except for the vehicle (5.0 μl of saline) injection, the remaining procedures were the same. After surgery, muscle and skin were sutured accordingly and penicillin (dosage and manufacture) was intramuscularly injected into the hind limbs. Those animals with surgical procedures were sent back to animal facility 30 min after completely waked up.

### 2.4 Blood pressure measurements

The non‐invasive systolic blood pressure (SBP, mmHg) and diastolic blood pressure (DBP, mmHg) of all rats were measured weekly using tail‐cuff method with a manometer‐tachometer (BP‐2010E; Softeron Biotechnology). Pulse pressure is equal to SBP minus DBP.^28^

### 2.5 Arterial baroreflex sensitivity

Following the previous protocol,^29^, ^30^ one cannula filled with heparin‐saline was inserted into the left femoral artery for BP and heart rate (HR) measurement through a transducer (model and manufacture) and another into the right femoral vein of the anesthetized rat (3% amobarbital sodium, 25 mg/kg, i.p.). The electrocardiogram was recorded (LabChart 7 Pro software; ADInstruments), and body temperature was maintained at 35°C. After postsurgical equilibration, 1, 3, and 10 μg/kg of sodium nitroprusside (SNP, Sigma) or phenylephrine (PE, Sigma) at incremental doses were injected intravenously to induce acute decreases and increases in BP, respectively. After each injection, the maximum change in HR at the peak change in mean arterial pressure (MAP) was recorded and ΔHR/ΔMAP was calculated as an index of baroreceptor gain.^31^

### 2.6 Blood pressure measurements while changing position in rats

To understand the effect of position (orthostatic or supine) on cardiovascular parameters, the MAP and HR, as well as baroreflex sensitivity, were collected under both orthostatic and supine position of anesthetic rats before and after DOPAL application.

### 2.7 Echocardiographic measurements

Trans‐thoracic echocardiography with an ultrasound machine (Vevo 2100 imaging system; VisualSonics) was used to test the cardiac functions (Table S1). Left ventricular systolic/diastolic internal diameter (LVIDs/LVIDd, mm), interventricular septum systolic/diastolic thickness (IVSs/IVSd, mm), and left ventricular systolic/diastolic posterior wall (LVPWs/LVPWd, mm) were measured simultaneously, and ejection fraction (EF, %) and fractional shortening (FS, %) were calculated from the short axis (SAX) or parasternal long axis (PSLAX)‐mode recording.

### 2.8 Transmission electron microscopy

All tissues were rapidly separated into a volume of about 1–2 mm^3^ and fixed in 2.5% glutaraldehyde in 0.1 mol/L PBS (PH 7.4). The specimens were then rinsed in buffer, post‐fixed in PBS‐buffered 1% OsO_4_ for 1–2 h, stained en bloc in uranyl acetate, dehydrated in ethanol, and embedded in epoxy resin by standard procedures. Then, the ultrathin slicer (UC‐7; Leica) was used for slicing, and the ultrathin sections were electron‐stained and observed under an electron microscope (JEM‐1220; JEOL Ltd.).

### 2.9 Quantitative real‐time polymerase chain reaction (qRT‐PCR)

Each sample of nodose (NG) or nucleus tractus solitarius (NTS), LVAW, or TA tissue was extracted from 2 or 1 rats, respectively, for both control (sham) and test (DOPAL) groups. All primers used (Table S2) were purchased from manufacturers, and the mRNA expression was determined using SYBR Green reagent in ABI 7500 Real‐Time PCR System (Applied Biosystems). Data of relative gene expression were analyzed with 2^−ΔΔCT^ method.

### 2.10 Western blot analysis

Each sample of NG or NTS, LVAW, and TA tissue was extracted from 3–4 rats or 1 rat, respectively. The total protein was incubated at 4°C for 1 h in RIPA buffer containing 1% protease inhibitor. The protein extracts were separated on 12% SDS‐PAGE and transferred to nitrocellulose membranes, which were further blocked with 5% non‐fat dry milk for 2 h, and then incubated at 4°C overnight with the primary antibodies anti‐GAPDH (Abcam Cat # G8795), anti‐β‐actin (Wanleibio, WL01845), anti‐tyrosine hydroxylase (Abcam, ab129991), and anti‐α‐synuclein (Abcam, ab27766), respectively, followed by incubation with the appropriate secondary antibodies (anti‐rabbit/anti‐mouse) at room temperature for 50–60 min. Specific antibody‐antigen complexes were detected using the Odyssey Infrared Imaging System (LI‐COR Biosciences).

### 2.11 Immunohistochemical analysis

The tissue slice (8 μm) of NG was prepared using the cryostat (LEICA cm 1850) and placed onto slide glass (2.5 × 8 cm) and fixed with pre‐cooled 4% paraformaldehyde and rinsed with phosphate‐buffered saline (PBS), and then incubated in PBS containing 1% BSA and 0.4% Triton X‐100 (Sigma) at 37°C for 1 h. After blocking in 10% normal goat serum at 37°C for 2 h, sections were incubated with primary antibodies for α‐synuclein and further incubated with TH at 4°C overnight. Fluorescence was observed under 594 and 488 spectrum, and selected images were taken using confocal microscope (Olympus Fluo‐view 300).

### 2.12 Statistical analyses

All data collected were tested for normality using Origin software (V. 7.0; Microsoft; descriptive statistics‐normality test: Shapiro‐Wilk), and the data that exhibit a normal distribution are before further evaluated for the statistical significance. The significant differences between the two groups were analyzed using two‐tailed unpaired Student's *t* test, while one‐way ANOVA followed by Bonferroni's post hoc test was also selected for multiple groups. Averaged pool data were expressed as mean ± SD. The *p* value < 0.05 was considered statistically significant.

## 3 RESULTS

### 3.1 Effects of DOPAL on BP and baroreflex sensitivity by vagal administration

**FIGURE 1:**
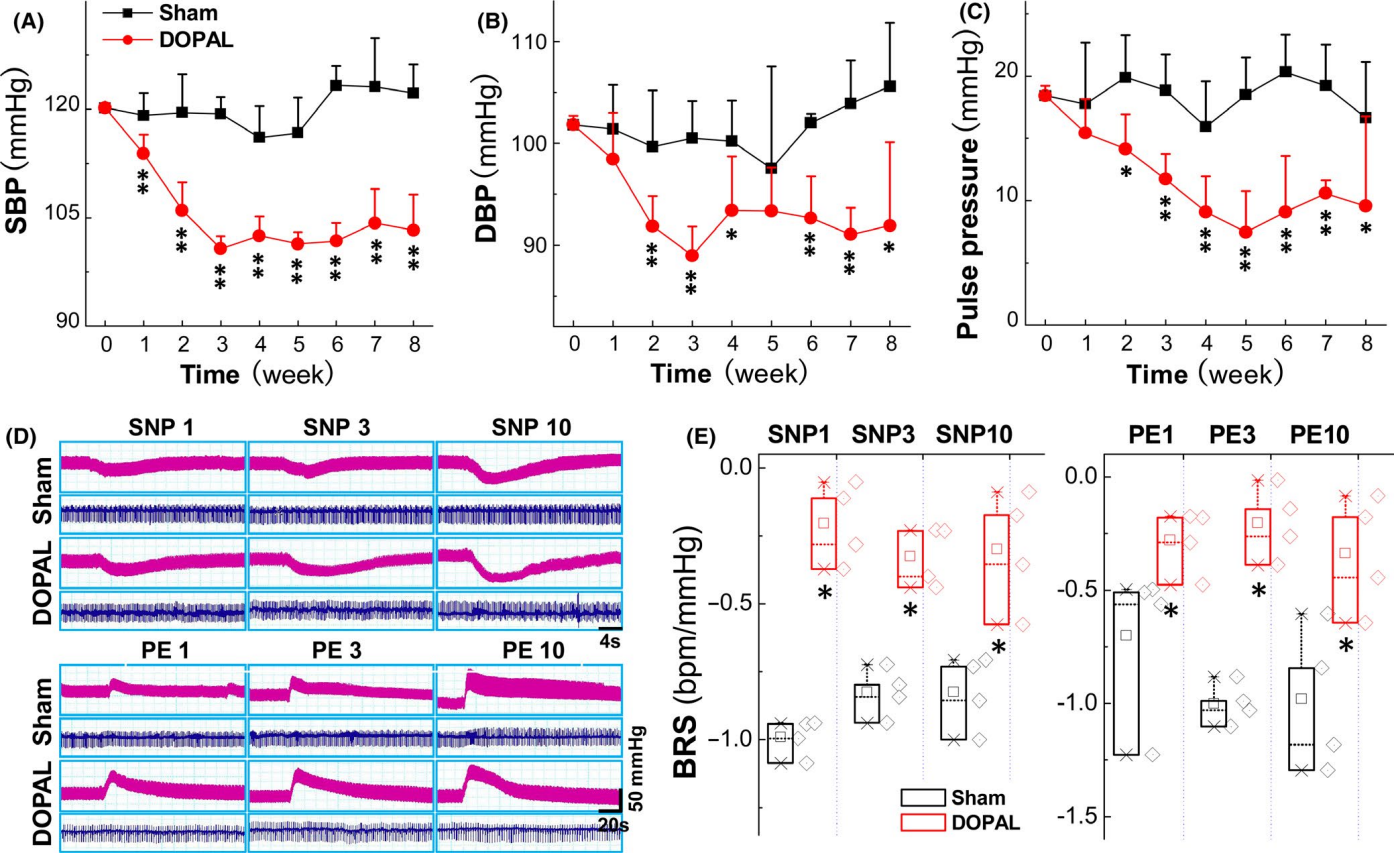
Changes in blood pressure and baroreflex sensitivity in rats administrated with DOPAL. (A–C) shows the systolic blood pressure (SBP), diastolic blood pressure (DBP), and pulse pressure difference, respectively, *n* = 6. (D, E) The representative changes in blood pressure (BP, pink traces) and heart rate (HR, blue traces) were monitored in the presence of 1, 3, and 10 μg/kg sodium nitroprusside (SNP) or phenylephrine (PE). The BRS (ΔHR/ΔMAP) was calculated before and after SNP and PE, *n* = 4. Data were expressed by mean ± SD. *p < 0.05 and **p < 0.01 versus sham

Based upon the hypothesis that the toxic product of dopamine‐DOPAL may be the key player in autonomic dysfunction through baroreflex afferent pathway, thereby DOPAL was microinjected into the vagus as described and BP, heart rates (HR), and baroreflex sensitivity (BRS) were monitored to evaluate the PD‐like autonomic dysfunction.^32^, ^33^, ^34^, ^35^ Compared with the sham group, SBP and DBP began to decrease at the first or second week and peaked at the third week after DOPAL administration and stayed stable status during observation (Figure 1A,B). The pulse pressure (pulse pressure is equal to SBP minus DBP) began to decrease at the second week to the lowest value at the fifth week and then restored partially (Figure 1C). Due to the importance of baroreflex in the rapid regulation of blood pressure, BRS, refers to the sensitivity of BP changes to reflex HR changes,^36^ is a good indicator for cardiac autonomic function, especially reflecting the tone of reflex vagus and sympathetic.^37^ So, the changes in BRS was also observed and the data (Figure 1D,E) showed that BRS was significantly reduced in DOPAL‐treated rats than that of the sham after administration of PE or SNP (1, 3, 10 μg/kg), suggesting that vagal administration of DOPAL‐mediated autonomic dysfunction is due at least partially to the impaired baroreflex function.

### 3.2 Alternations of BP while changing position in control and DOPAL‐treated rats

**FIGURE 2:**
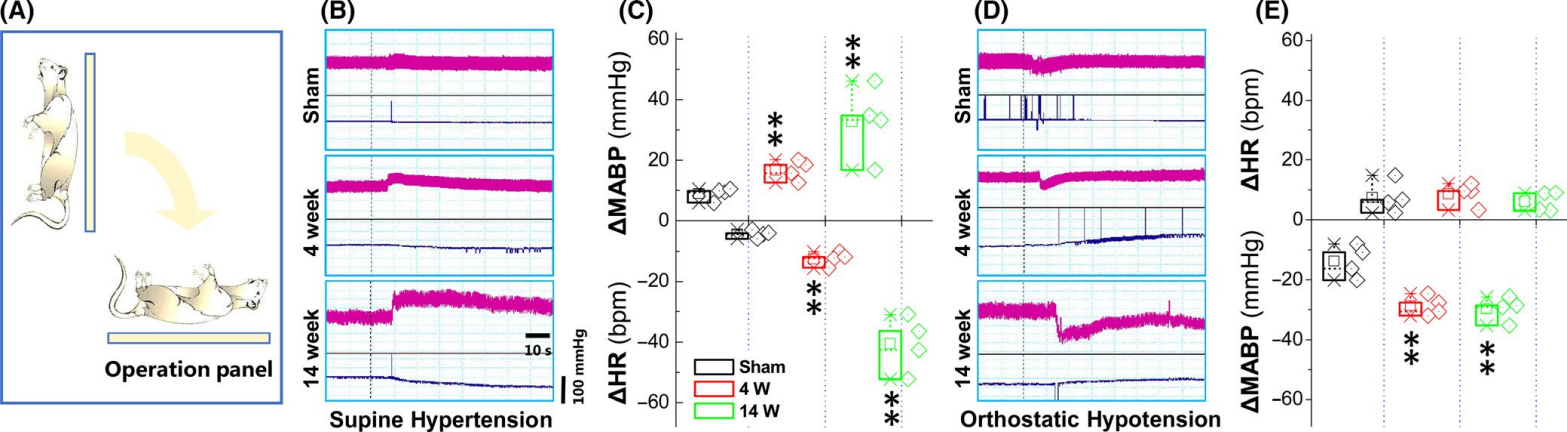
BP changes while changing position in rats administrated with DOPAL. (A) Schematic diagram of experimental operation. Rats were fixed on the operating table, and the heart rate and blood pressure at orthostatic or supine status were recorded in real time using femoral artery cannulation. (B–E) The representative changes in blood pressure (BP, pink traces) and heart rate (HR, blue traces) at orthostatic or supine status compared with steady state, and the differences were expressed as mean ± SD. **p < 0.01 versus sham, *n* = 4

Large body evidence has documented that the earlier autonomic failure manifests clear OH and/or SH in the PD patients^6^, ^38^, ^39^ that is the major cause of cardiovascular events and fractures, even to death in PD patients. To simulate clinical OH and SH in the current model, we tested the changes in BP while changing the position in an unconscious rats with or without vagal application of DOPAL. Notably, the results showed that, compared with the sham group, when placing the rats at horizontal position (Figure 2A) a significant and time‐dependent increase in the mean arterial BP (MABP) and recover time were both confirmed along with a time‐dependent decrease in HR (Figure 2B,C). In stark contrast, exact opposite phenomena were observed with no changes in HR (Figure 2D,E) when placing the rats at vertical position, indicating that the current model successfully simulates clinical OH and SH. Surprisingly, a time‐dependent increase in BP at SH position companies with significant decrease in HR, suggesting that parasympathetic innervation remains intact and the negative chronotropic action could be achieved by acetylcholine released from the terminal; however, when placed rats at OH position, the BP went down while HR was not altered simultaneously, indicating that the sympathetic terminal to the heart is denervated with less amount of noradrenaline released.^40^, ^41^

### 3.3 Changes in cardiac function and ultrastructure of myocardium in control and DOPAL‐treated rats

**FIGURE 3:**
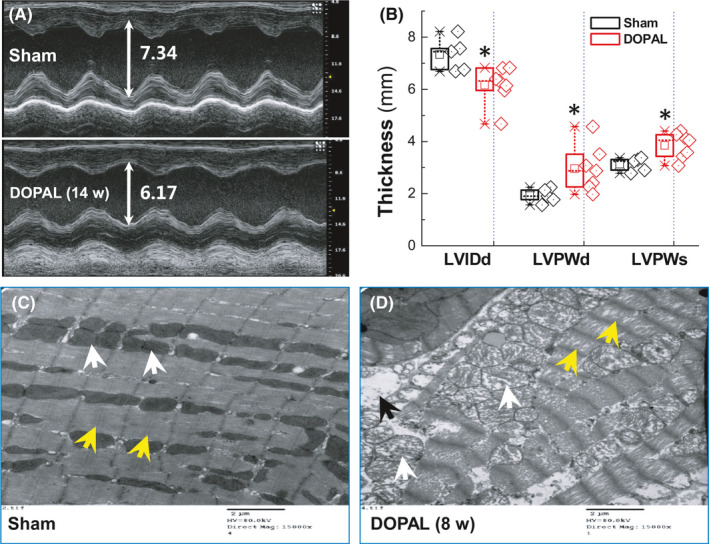
Ultrasound results of DOPAL‐administered rats and representative images of transmission electron microscope. (A, B) Ultrasonic evaluation of cardiac function during DOPAL application, LVIDd: left ventricular diastolic internal diameter, LVPWd/s: left ventricular diastolic/systolic posterior wall. Data were presented as mean ± SD. *p < 0.05 versus sham. *n* = 5–7; (C, D) Representative images of transmission electroscope of LVAW from rats at 8 w after DOPAL administration. Scale bar: 2 μm, direct Magnification: 15,000×, the yellow arrowheads: filaments of myocardium, white arrowheads: mitochondria, and black arrowheads: autophagic vesicles

Studies have shown that autonomic dysfunction often involves the heart and leads to cardiovascular dysfunction.^42^, ^43^ To this end, echocardiography and electron microscopy were conducted to evaluate cardiac function and corresponding changes in ultrastructure. Echocardiographic data showed that, compared with the sham group, the left ventricular diastolic inner diameter (LVIDd) was significantly reduced (Figure 3A,B) along with obvious thickening of the LVPWd and LVPWs at 14 w after DOPAL administration, indicating that the rat heart is in a compensatory state to losing sympathetic support and/or hypotension (OH). In the meantime, the ultrastructure of LVAW was observed under transmission electron microscope and the results (Figure 3C,D) showed that the mitochondria were swollen, the filaments of myocardium were loosened or even broken, and the autophagic vesicles could be visible at 8 w after DOPAL administration compared with the sham group. These data strongly suggest that cardiac histomorphology is abnormal, mainly manifested by impaired energy metabolism and systolic function due presumably to α‐Syn aggregation induced by DOPAL exocytosis from presynaptic membrane. In addition, the ultrastructure of the thoracic aorta (TA) was also observed under the similar experimental condition and found that the endothelial cells appeared to fall off over time and completely fell off at 10 w after DOPAL treatment (Figure S1), implying DOPAL‐mediated denervation could also be seen on aorta.

### 3.4 Expression profiles of α‐Syn in LVAW and TA of control and DOPAL‐treated rats

**FIGURE 4:**
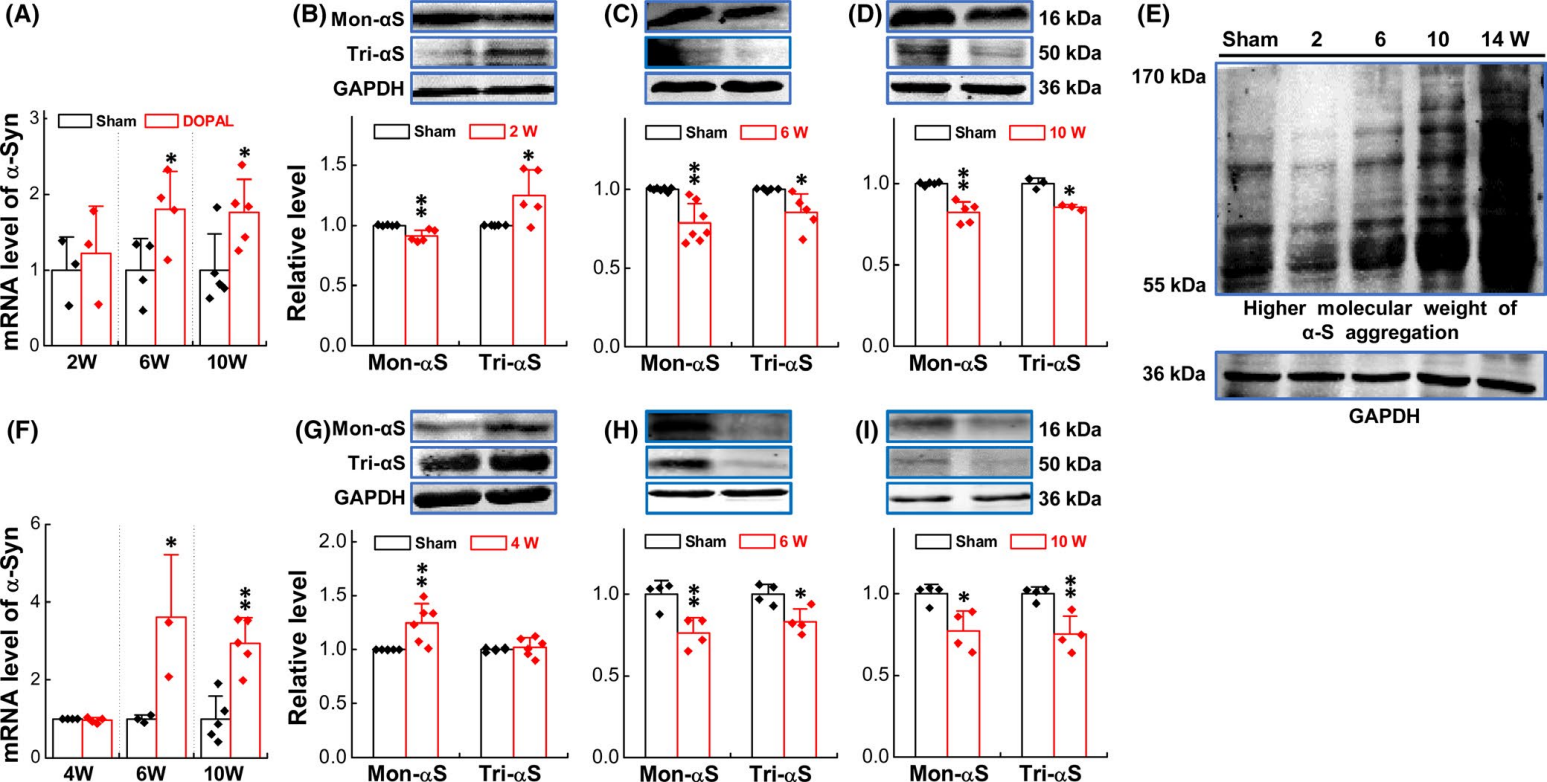
Expression of α‐Syn in LVAW and TA. (A) mRNA expression of α‐Syn in LVAW at different time points; (B–D) protein expression of α‐Syn in LVAW at different time points; (E) higher molecular weight of α‐Syn aggregation in LVAW; (F) mRNA expression of α‐Syn in TA at different time point; and (G–I) protein expression of α‐Syn in TA at different time point. Data were expressed as mean ± SD. *p < 0.05 and **p < 0.01 versus sham, *n* = 3–5

Functional and morphological data all pointed out that vagal administration of DOPAL somehow impaired cardiac function with morphological alternation, suggesting that DOPAL is likely to be transported to the heart via vagal efferent causing local denervation found in PD patients.^44^ Notably, this hypothesis is consistent with the notion that α‐Syn aggregated in distal axons is disappeared with the disappearance of dopaminergic axons, while accumulates more in paravertebral sympathetic ganglia.^45^ Based on this, we speculate that after application of DOPAL on vagus, DOPAL could be transported presumably through vagal efferent to the heart and induce α‐Syn aggregation leading to the toxic effect on nerve terminal and innervated cardiac tissue. To verify it, we first examined the expression of α‐Syn in LVAW and TA (Figure 4A,F). These data showed that the levels of α‐Syn mRNA in LVAW and TA were significantly up‐regulated compared with the sham group over time. Inconsistently, in LVAW at 2 w after vagal application of DOPAL, the expression of Mon‐α‐Syn and Tri‐α‐Syn was significantly down‐regulated and up‐regulated, respectively (Figure 4B), whereas, at 6 w after vagal application of DOPA, the expression of both Mon‐α‐Syn and Tri‐α‐Syn showed significant trend of down‐regulation (Figure 4C) and this trend was further strengthened at 10 w after vagal application (Figure 4D). In TA (Figure 4G–I), at 4 w after vagal application of DOPAL, up‐regulated Mon‐α‐Syn was more dramatic than that of Tri‐α‐Syn compared with sham control (Figure 4G). And the expression of both Mon‐α‐Syn and Tri‐α‐Syn showed significant trend of down‐regulation at 6 and 10 w (Figure 4H,I). In stark contrast, inversely down‐regulated Mon‐α‐Syn and Tri‐α‐Syn were confirmed at both 6 w and 10 w after vagal application of DOPAL. These results have clearly demonstrated that abnormal expression of α‐Syn is detected at both LVAW and TA, and the expression pattern from higher to lower expression strongly suggests that monomers of α‐Syn are likely to aggregate and shift to higher molecular weight of α‐Syn oligomers over time (Figure 4E).

### 3.5 DOPAL‐mediated ultrastructure changes in the NG and NTS

**FIGURE 5:**
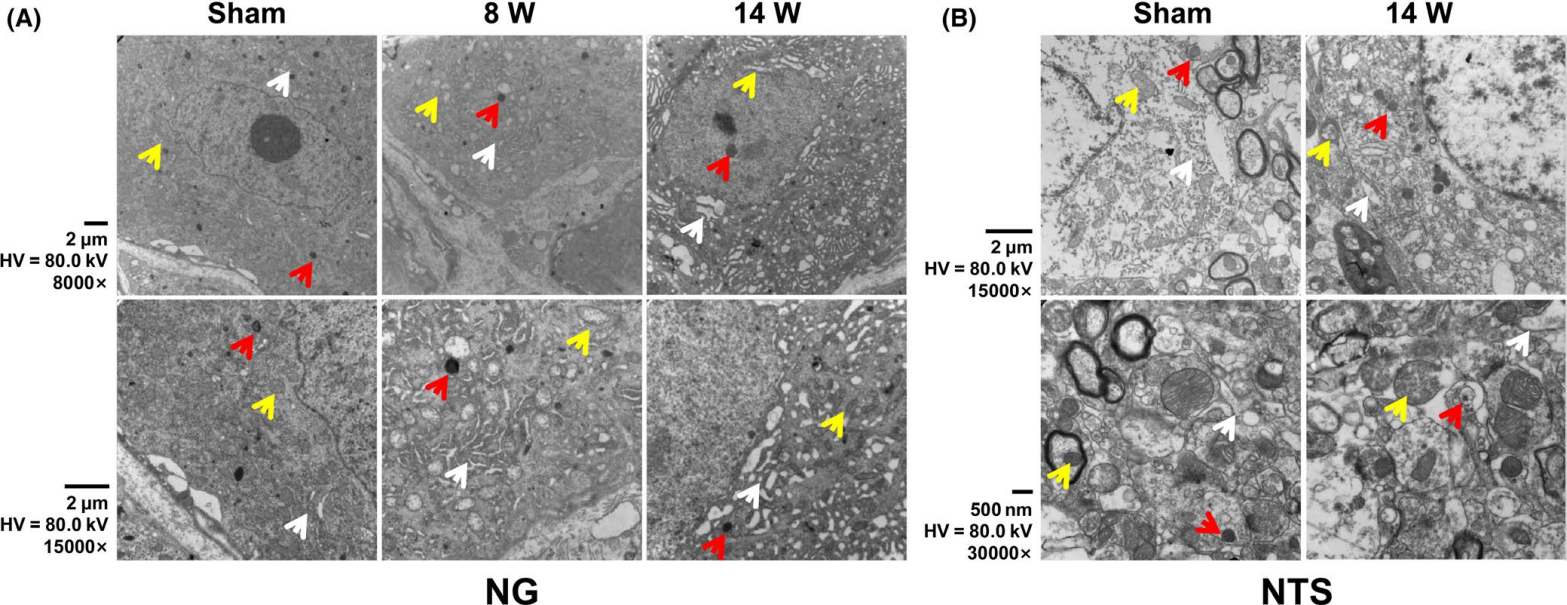
Representative images of transmission electron microscope. (A) The representative image of NG in rats administrated with DOPAL. (B) The representative image of NTS. Scale bar: 2 μm and 500 nm, direct Magnification: 8000× and 15,000×, the yellow arrowheads: mitochondria, white arrowheads: endoplasmic reticulum, and red arrowheads: lysosome

We have demonstrated that arterial baroreflex function is impaired after vagal application of DOPAL. The NG and NTS are important components of the baroreflex afferent pathway.^46^, ^47^ Are they involved in this pathological process and how do they change morphologically? To answer this particular question, the ultrastructure of NG and NTS was observed under transmission electron microscope. The results showed that, compared with the sham group, both mitochondrial and endoplasmic reticulum swellings and hyperplasia were confirmed in a time‐dependent fashion with the number of lysosome increased in the NG after vagal application of DOPAL and the longer the administration time, the more serious the organelle damage (Figure 5A). A similarity of DOPAL‐mediated injury was also observed in the NTS (Figure 5B).

### 3.6 Expression profiles of α‐Syn in the NG and NTS of control and DOPAL‐treated rats

**FIGURE 6:**
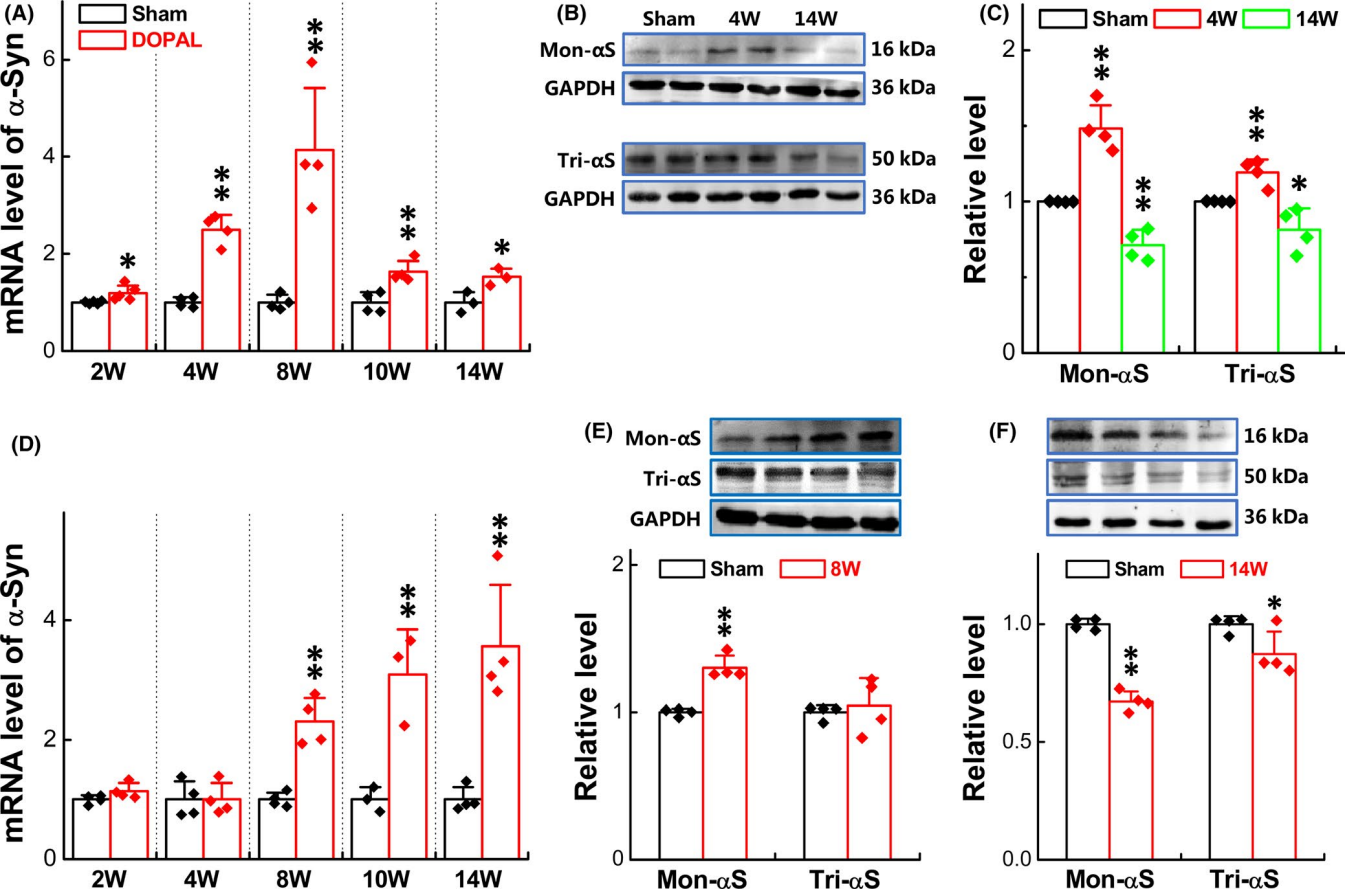
Expression of α‐Syn in the NG and NTS. (A) mRNA expression of α‐Syn in the NG at different time points; (B, C) protein expression of Mon‐ and Tri‐α‐Syn in the NG at 4 and 14 w; (D) mRNA expression of α‐Syn in NTS at different time point; and (E, F) protein expression of Mon‐ and Tri‐α‐Syn in the NTS at 8 and 14 w. Data were presented as mean ± SD, *p < 0.05 and **p < 0.01 versus sham, *n* = 3–5 from 12 to 16 rats

As a biomarker of Parkinson's disease, α‐Syn plays an important role in the pathological process of PD.^48^ Studies have reported that α‐Syn will produce neurotoxic damage to dopaminergic neurons after misfolding or aggregation.^49^, ^50^ Combined with the morphological alternation of NG and NTS, we would like to further explore the expression changes of the α‐Syn in these two parts. The PCR results showed that the mRNA expression of α‐Syn in the NG increased time‐dependently after vagal application of DOPAL and decreased until 8 w, but it remained at higher level than the sham group (Figure 6A); immunoblotting results showed that the expression of Mon‐α‐Syn and Tri‐α‐Syn increased at 4 w and decreased at 14 w after vagal application of DOPAL (Figure 6B,C). Whether or not this transition means the transformation from lower to higher molecular weight of Syn is our next question. As expectation, we detected the expression of higher molecular weight of α‐Syn at different time periods in the NG by Western blot and the data showed that α‐Syn indeed extends to higher molecular weight over time (Figure S2 left panel).

In the case of NTS, the mRNA expression of α‐Syn was significantly time‐dependently increased at the 8 w up to the end of observation (14 w) after vagal application of DOPAL (Figure 6D). So, immunoblotting was also started at the same time and the results showed that Mon‐α‐Syn was increased without significant change in Tri‐α‐Syn at 8 w (Figure 6E), both of them were significantly down‐regulated at 14 w (Figure 6F) after vagal administration of DOPAL, implicating that Mon‐ and Tri‐α‐Syn aggregate to higher molecular weight of α‐Syn over time and this speculation was then confirmed (Figure S2 right panel).

### 3.7 Relationship between α‐Syn aggregation and TH in the NG

**FIGURE 7:**
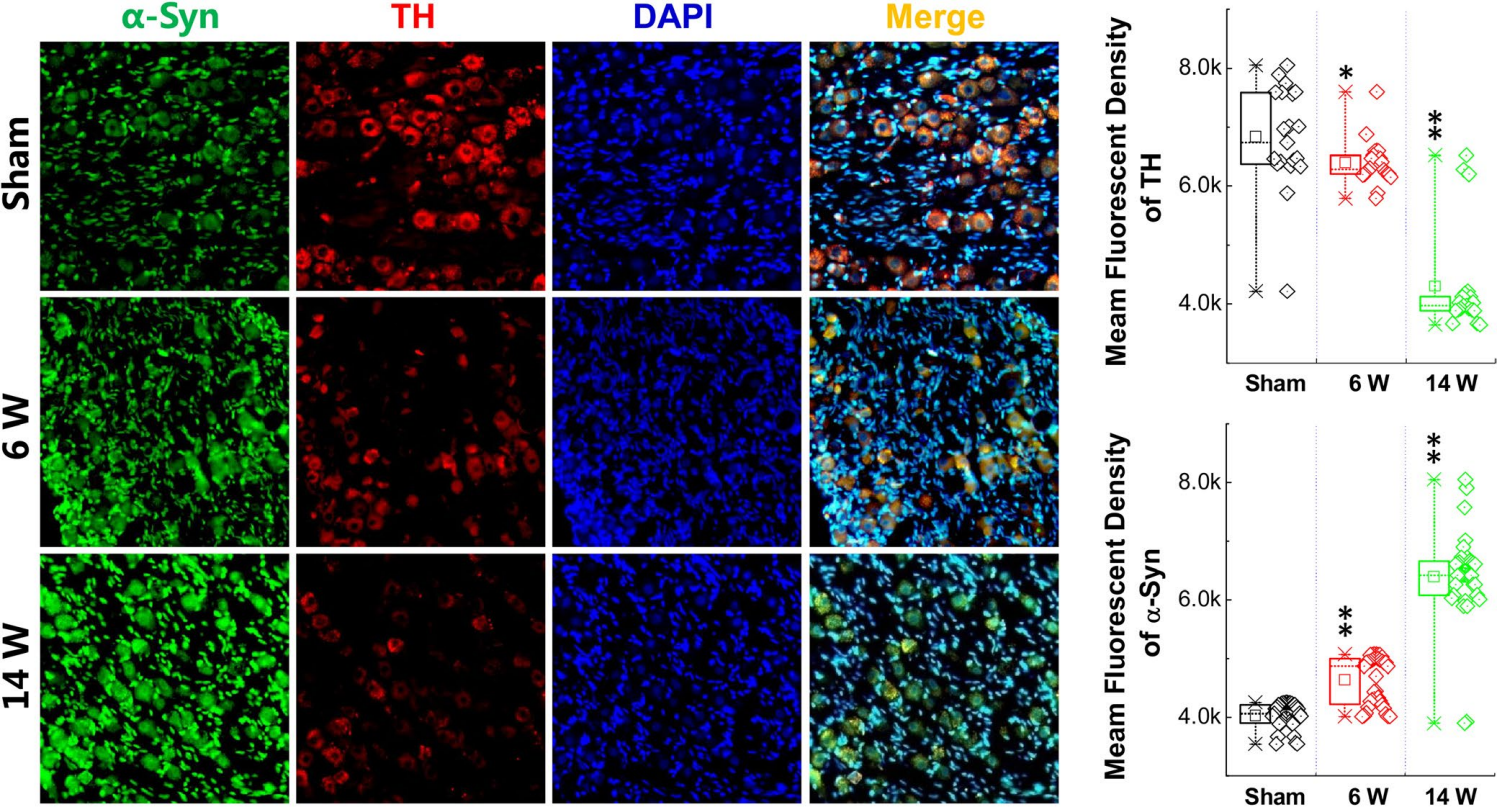
Expression of α‐Syn and TH in the NG. Left panel: Representative graph of immunohistochemistry (IHC) results of α‐Syn at NG in rats administrated with DOPAL. Right panel: Immunohistochemical quantitative statistics. Data were presented as mean ± SD, *p < 0.05 and **p < 0.01 versus sham, *n* = 20–35

Tyrosine hydroxylase (TH) is a key enzyme in dopamine synthesis and if α‐Syn aggregation affects the function of TH, consequent and correspondent changes in TH expression of neurons in the NG are expected along with α‐Syn aggregation after vagal application of DOPAL. To this end, the co‐localization of α‐Syn and TH detected with specific antibodies was conducted (Figure 7 left panel) and the results showed that either TH or α‐Syn was expressed in the cell membrane and cytoplasm and the fluorescent intensity for TH (Figure 7 upper right) and α‐Syn (Figure 7 lower right) was markedly decreased and increased, respectively, in a time‐dependent manner. Coefficient of correlation index (r2) equals to 0.996, suggesting a close relationship of changing between α‐Syn and TH in the NG. Even though a dramatic α‐Syn aggregation occurred at 14 w after vagal administration of DOPAL, the number and integrity of neurons with TH‐positive labeling remained, implying the inhibitory effect of α‐Syn aggregation on TH function.^41^, ^51^

## 4 DISCUSSION

The major contributions of the current study are as follows: (1) experimentally and evidently mimicking the early PD‐like autonomic dysfunction, such as OH and SH, by means of vagal administration of DOPAL and pointing out a plausible peripheral mechanisms of PD; (2) vagal application of DOPAL not only impairs the baroreflex function, but also alters LVAW and TA morphology, pointing out a potential involvement of degeneration in the nerve endings innervated to the heart and aorta, with which DOPAL and/or DOPAL‐mediated higher molecular weight of α‐Syn can be transported via vagal efferents down to its innervated organs; (3) DOPAL promotes time‐dependent α‐Syn formation in the NG and NTS, and the expression patterns reveal the toxic effect of aggregated α‐Syn from lower to higher molecular weight of α‐Syn over time, pointing out the transportation of DOPAL and/or DOPAL‐mediated higher molecular weight of α‐Syn via vagal afferents up to NG and NTS; and (4) the correlation change in α‐Syn and TH is the most likely reason to further reduce dopamine synthesis and this pathological process in turn exerts additional inhibition of TH function by α‐Syn aggregation, which worsens the deficiency of dopamine and further impairs the PD‐like autonomic dysfunction.

The evidence from this investigation definitely extends our current understanding of the etiology of PD that may initiate from the peripheral site due presumably to an unusual metabolism of DA and accumulation of DOPAL, consequently mediating α‐Syn aggregation and causing further dysfunction of central dopaminergic system through toxic effect of DOPAL and α‐Syn on axonal transportation, eventually causing an early autonomic dysfunction of BP regulation. This autonomic failure of BP regulation of PD is strongly supported by recent finding that lower urinary track and gastrointestinal dysfunction are common in early PD^52^, ^53^, ^54^ and these non‐motor deficits of during early PD development such as autonomic dysfunction are well described in a recent review article.^55^ The better understanding of early autonomic dysfunction and underlying mechanisms is definitely required to offer better therapeutic options to PD patients.^56^, ^57^

To achieve the goal of the current study, we successfully attempted to establish a new technique of vagal administration of DOPAL and by using this technique the changes in BP and baroreflex function were collected for simulating early PD‐like early autonomic dysfunction. More importantly, also by using this technique, the BP changes while changing the body position of the tested rats were confirmed, manifested as significant orthostatic OH and SH compared with sham control, and this technique paves the way for our following experimentation, not only changed BP and cardiac function, but also altered LVAW and TA morphology in a time‐dependent fashion after vagal application of DOPAL. These observations strongly suggest that vagal DOPAL mediated toxic effects via promoting α‐Syn formation from lower to higher molecular weight presumably through vagal efferents and afferents, respectively, to their innervated organs. Notably, this hypothesis is fully supported by the facts that Mon‐, Tri‐, and even higher molecular α‐Syn are clearly detected in the heart and TA (efferents) and in the NG and NTS (afferents) time‐dependently.

The functional study found that the BP and pulse pressure were significantly changed (Figure 1A–C) and echocardiographic measurements (Figure 3A,B) showed a compensatory wall thickening after application of DOPAL, which is due at least partially to sympathetic nerve degeneration and/or losing TH‐mediated catecholamine production. Importantly, functional alternations were evidently supported by our ultrastructure observations in LVAW slices (Figure 3C,D) showing a series of pathological changes, including mitochondrial swelling, myofilament rupture, and transient autophagy.

It has been demonstrated in the past that in almost all PD patients with OH, neurochemical, neuroimaging, and neuropharmacological studies have also shown that cardiac and extra‐cardiac sympathetic noradrenergic denervation histopathologically confirmed^41^, ^58^ with lower baroreflex sensitivity. Meanwhile, the study on PD patients has shown cardiac sympathetic denervation with failure to increase total peripheral resistance leading to a large reduction in SBP than control group under orthostatic stress test.^43^ These all seem to indicate that cardiovascular system dysfunction of PD is closely related to cardiovascular denervation. Surprisingly, their observations were consistent well with the data generated from the animal experiments using our newly designed techniques, that is, monitoring BP while changing the body position after vagal application of DOPAL, with which, the pressure reflex function (Figure 1C,D) was damaged with severed OH and SH in DOPAL‐treated rats in a time‐dependent fashion (Figure 2), which all conform to the pathological changes in PD‐like autonomic dysfunction. As expected, PD‐like OH and SH are largely attributed to the toxic effect of DOPAL on baroreflex afferent functions, which is a key reason to impair the adaptation through re‐adjustment of baroreceptor sensitivity. This impairment was firstly been confirmed by the ultrastructure showing tremendously morphological alternations in the NG and NTS region (Figure 5) after vagal administration of DOPAL.

After application of DOPAL on vagus nerve, how did DOPAL produce toxic effects on both the nervous and circulatory system, which may be related to a transient increase in the concentration of DOPAL caused by local injection, making the aggregation of α‐Syn. The evidence has shown that DOPAL promotes α‐Syn monomer aggregation^59^ that serves as self‐template to “infect” healthy α‐Syn monomers to form higher molecular weight of α‐Syn.^60^ This may be a key molecular mechanism for the formation of α‐Syn aggregation in the heart‐TA and NG‐NTS after vagal application of DOPAL. Firstly, our functional and morphological changes have been confirmed on both efferent (heart and TA) and afferent (NG and NTS) sides of microinjection, and DOPAL‐mediated molecular alternations would be not surprised. Following this direction, the tests of gene and protein expression with various molecular weights of α‐Syn clearly demonstrated a time‐dependent transformation of Mon‐ and Tri‐α‐Syn to higher molecular form of toxic oligomers in the heart‐TA and NG‐NTS (Figures 4, 6 and 7). And evidences also confirm the transportation of DOPAL from application site down and up to the heart and NG‐NTS via vagal efferents and afferents, respectively.

Taken together, the current investigation develops a new technique, by which a toxic DOPAL, a toxic metabolic of dopamine, can be microinjected in the vagus to simulate the PD‐like early autonomic dysfunction, including OH and SH, while changing the body position after vagal application of DOPAL. The advantage is absolutely obvious, but there are also some limitations. On the one hand, after application of DOPAL on vagus, there is no clue whether DOPAL immediately promotes α‐Syn aggregation locally at injection site, or plays the role after it is transported via axon flow to the heart and TA via vagal efferent fibers, or to the NG and NTS via afferent fibers; therefore, further study is definitely needed. On the other hand, it is not clear how aggregated α‐Syn is transported during this process. Additionally, whether or not vagal application of DOPAL can cause sympathetic/parasympathetic denervation/degeneration at organ level (heart/aorta) needs to be identified under the current experimental condition even though it has been observed in PD patients.^44^, ^61^ Therefore, these remained questions will be our focuses of ongoing project. PD is a serious neurodegenerative disease, so seeking biomarkers have always been the goal pursued by scientists and finding right animal model to mimic an early PD‐like autonomic dysfunction has important clinical implications for the prevention and treatment of Parkinson's disease.

## 5 CONCLUSION

**FIGURE 8:**
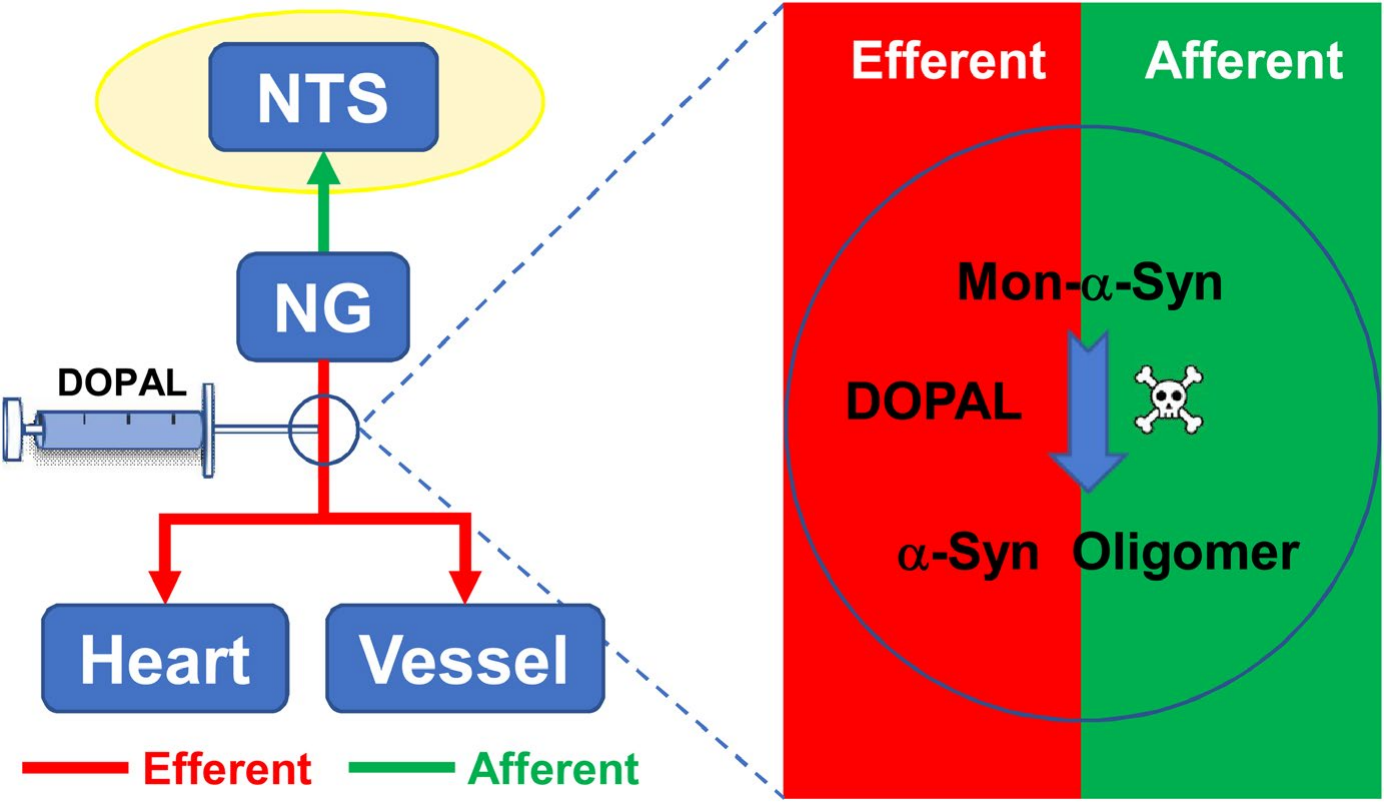
Schematic diagram of DOPAL administration. Application of DOPAL on vagus causes significant accumulation of α‐Syn monomers to form toxic oligomers that could be transported to the heart‐TA and NG‐NTS by axon flow

In brief, we propose that (Figure 8), after application of DOPAL on vagus, a brief increase in DOPAL causes significant accumulation of α‐Syn monomers in the vagus to form toxic oligomers that could be transported to the heart‐TA and NG‐NTS by axon flow leading to autonomic dysfunction.

## CONFLICT OF INTEREST

The authors declare that there is no conflict of interest associated with the contents of this article.

## Supporting information

Supplementary Material

## Data Availability

The data that support the findings of this study are available from the corresponding author upon reasonable request.
