# Supplementary material for: Parkinson‐like early autonomic dysfunction induced by vagal application of DOPAL in rats
Source: CNS Neurosci Ther. 2021 Jan 21;27(5):540–51. doi: 10.1111/cns.13589 (PMC8025611; doi:10.1111/cns.13589)
Supplement: Supplementary file 1 — Supplementary Material [file CNS-27-540-s001.doc]

**Supplemental Material**

**Parkinson-like early autonomic dysfunction induced by vagal application of DOPAL in rats**

Jie Sun^1,2,†^, Chao He^1.2,†^, Qiu-Xin Yan^1.3^, Hong-Dan Wang^1^, Ke-Xin Li^1,4^, Xun Sun^1,4^, Yan Feng^1,4^, Rong-Rong Zha^1,4^, Chang-Peng Cui^1^, Xue Xiong^1^, Shan Gao^1^, Xue Wang^1^, Rui-Xue Yin^1^, Guo-Fen Qiao^1^, Bai-Yan Li^1^,*

^1^Department of Pharmacology (State-Province Key Laboratories of Biomedicine-Pharmaceutics of China, Key Laboratory of Cardiovascular Medicine Research, Ministry of Education), School Pharmacy of Harbin Medical University, Harbin, China

^2^School of Pharmaceutical Science, Sun Yat-Sen University, Shenzhen, China

^3^School of Life Science and Technology, Harbin Institute of Technology, Harbin, China

^4^Department of Biomedical Engineering, School of Engineering and Technology, Indiana University Purdue University Indianapolis, Indianapolis, USA

*Corresponding to: Bai-Yan Li, Department of Pharmacology, Harbin Medical University, #157 Baojian Road, Harbin 150081, China; Tel:/Fax: +86(0)451-8667-1354,

E-mail: [liby@ems.hrbmu.edu.cn](mailto:liby@ems.hrbmu.edu.cn) for B.-Y.L.

^†^These authors contributed equally to this work.

**Supplemental Tables:**

**Table S1: Effect of DOPAL administrated by vagal microinjection on cardiac parameters ultrasound data.** All data are presented as mean ± SD. **P*  0.05 *vs.* Sham.

| **Parameters** | **Sham** | **DOPAL** |
| --- | --- | --- |
| IVS; d-D mm | 2.11 ± 0.32 | 2.34 ± 0.42 |
| IVS; s-D mm | 3.16 ± 0.27 | 3.28 ± 0.57 |
| LVID; d-D mm | 7.34 ± 0.63 | 6.17 ± 0.73* |
| LVID; s-D mm | 4.25 ± 0.65 | 3.57 ± 0.67 |
| LVPW; d-D mm | 1.92 ± 0.27 | 2.95 ± 0.88* |
| LVPW; s-D mm | 3.12 ± 0.26 | 3.85 ± 0.49* |
| EF % | 69.98 ± 8.13 | 70.14 ± 7.34 |
| FS % | 40.72 ± 6.09 | 40.6 ± 5.9 |
| LV Vol μl | 286.12 ± 55.59 | 184.32 ± 58.48* |

Note: EF, ejection fraction; FS, fractional shortening; IVSs/IVSd, systolic/diastolic left ventricular septum thickness; LVIDs/LVIDd, systolic/diastolic left ventricular internal diameter; LVPWs/LVPWd, systolic/diastolic left ventricular posterior wall.

**Table S2: All primers used in the experiments are listed.**

| **Primers** | **Sequence** |
| --- | --- |
| GAPDH | Fwd：5'-AAGAAGGTGGTGAAGCAGGC-3' |
|  | Rev：5'-TCCACCACCCAGTTGCTGTA-3' |
| α-Syn | Fwd：5'-GAGGGAGTCGTTCATGGAGT-3' |
|  | Rev：5'-CATTTGTCACTTGCTCTTTGG-3' |

**Supplemental Figure 1:**


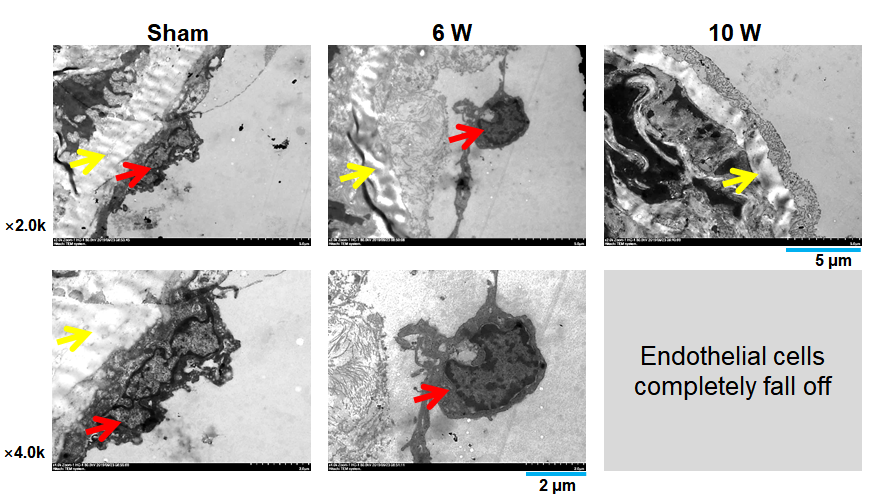


**Figure S1: Representative images of electron microscope of TA.** The representative image of TA in rats administrated with DOPAL at 6 w and 10 w. Scale bar: 5 μm and 2 μm applied for other images on the same row. Direct Magnification: 2.0K×, 4.0K×. The yellow and red arrowheads mean elastic layer and endothelial cells, respectively.

**Supplemental Figure 2:**

**
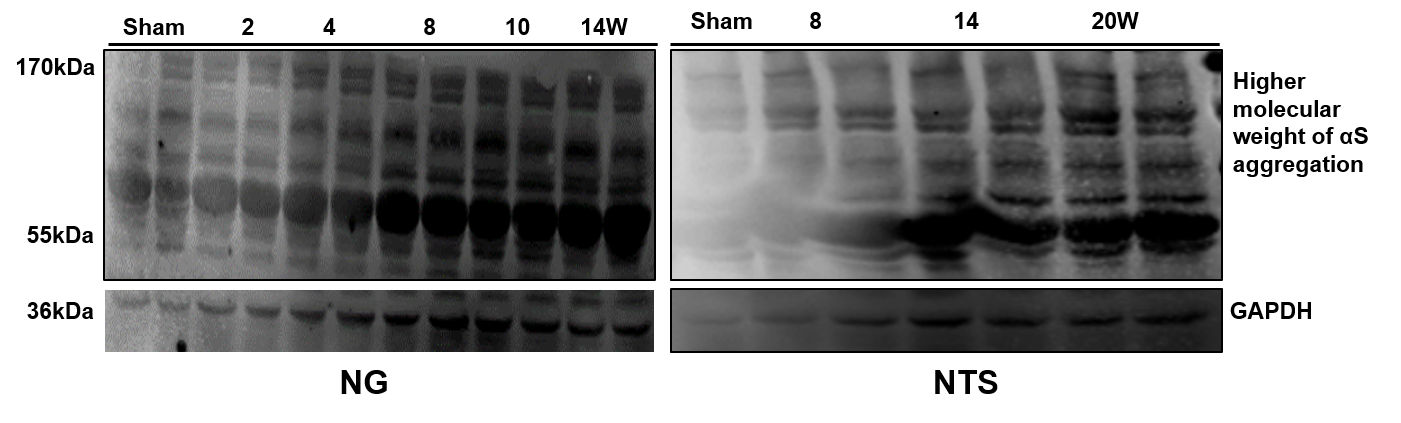
**

**Figure S2: Higher molecular weight of α-Syn aggregation in the NG and NTS. Left panel**: Protein expression of α-Syn in the NG at different time point; **Right panel**: Protein expression of α-Syn in the NTS at different time point.
